# Anterior Poliomyelitis, or Infantile Paralysis
*A paper read at the meeting of the Bath, Bristol and Somerset Branch of the British Medical Association on 25th January, 1933.


**Published:** 1933

**Authors:** H. Chitty

**Affiliations:** Surgeon to the Bristol Royal Infirmary and to Winford Orthopædic Hospital; Consulting Surgeon to the Royal Hospital for Sick Children and Women, Bristol


### ANTERIOR POLIOMYELITIS, OR INFANTILE PARALYSIS.*

BY

H. CHITTY, M.S., F.R.C.S.,

*Surgeon to the Bristol Royal Infirmary and to Winford Orthopædic Hospital;*
*Consulting Surgeon to the Royal Hospital for Sick Children and Women, Bristol.*

---

THE subject of infantile paralysis is of wide general interest, since the treatment of almost every case calls for the co-operation of the general practitioner with physician, surgeon, pathologist and public health officer. The disease has been known for a long time, having first been described by an Englishman, Michael Underwood, in 1784.

Of late years the annual notifications in this country have varied between a minimum of 386 in 1922 and a maximum of 1,396 in 1926. I am convinced that the disease is much more common than these figures would suggest, and that many of the milder cases remain undiagnosed while yet more escape notification.

Great Britain has been lucky to have escaped any serious epidemics such as those which have afflicted other countries. The greatest of these attacked New York City and State in 1916, when there were 13,223 cases and the mortality exceeded 25 per cent. In the same area more than 2,000 cases were reported

* A paper read at the meeting of the Bath, Bristol and Somerset Branch of the British Medical Association on 25th January, 1933.

in 1931. Other notable epidemics have taken place in recent years in Scandinavia, New Zealand, Australia and Central Europe. As we can never tell when our own luck may turn, it is just as well that we should strive to learn all that we can of modern methods of prevention, diagnosis and treatment. It is from the intensive study of poliomyelitis during epidemics that most of the advances in our knowledge have been derived. A private benefaction to the State of Vermont, U.S.A., enabled most valuable experimental and clinical work to be carried out from 1910 onwards, and in connection with this the names of Caverley, Flexner and Lovett became justly famous.

I do not propose in this paper to dwell upon details of surgical treatment, but rather to attempt a sketch of anterior poliomyelitis from a general standpoint. In so doing I shall trespass upon the domains of other branches of the profession, but as my excuse I must plead that the provinces of physician and surgeon overlap at most stages of the disease, and that each must strive to know something of the other's territory.

As you all know, infantile paralysis crops up sporadically at all times of the year, though most commonly in the late summer and the autumn. It is also during these months that epidemics usually arise. During the first six months of life natural immunity is high, though I have come across the record of one case commencing in a child aged six weeks. The highest incidence is during the second year of life, and the vast majority of cases arise during the first decennium. From then onwards there is a rapid fall, though persons may be attacked at any time of life. In persons over twenty years of age the mortality is very high, frequently 50 per cent. or more. Death is most commonly due to involvement of the muscles of respiration. It is a curious fact that all epidemics show a slight preponderance of males amongst those attacked.

The only animal in which the disease can be experimentally produced is the monkey. In several instances the virus, obtained from human sources, has been experimentally passed through a series of monkeys. Infection usually takes place through the nose, and in monkeys which have been originally infected by direct inoculation into the brain or into the peritoneum the virus has been subsequently isolated from the nasal mucus. It is known to be ultra-microscopic, since it will pass through a Berkefeld filter V though it is held up by a Berkefeld W. The incubation period of the disease is commonly three or four days, but it may vary from two to ten.

Acute poliomyelitis is a generalized infection, and some changes are found in most of the organs of the body and in the lymphatic tissues, but the brunt of the attack is borne by the nervous system. In experimentally produced cases one of the earliest changes found is swelling and hyperæmia of the posterior root ganglia. This is of interest, since hyperæsthesia, either general or confined to one or more limbs, is one of the most constant symptoms during the acute stage of the disease. Meningitis arises early. There is hyperæmia and œdema of the cord associated with perivascular infiltration and often hæmorrhages. The grey matter of the anterior horns suffers most, and the lumbar enlargement is more commonly and extensively involved than other parts of the cord. The motor cells of the anterior horns may be irreparably destroyed or they may be only temporarily thrown out of action by the pressure of blood and exudate. It is owing to this latter fact that some degree of recovery from the initial paralysis may always be expected.

The clinical types are various. One sometimes meets with a fulminating type in which paralysis is the first thing noticed, and in which the child may be very ill with every symptom of a severe generalized infection. Much more commonly, however, the patient appears to have a feverish cold with sore throat, running nose and a rise of temperature. An initial attack of vomiting is very common, and there may be diarrhœa, retention of urine, and sometimes a rash. The rash is not distinctive, and may be mistaken for German measles or scarlet fever. Unless an epidemic happens to be raging at the time, the child will probably be thought to be suffering from an attack of influenza. The first stage may pass directly into the second in which meningeal symptoms appear, but there may be a short interval during which the child appears to have recovered, and may even go back to school. After this brief intermission the temperature rises again. This variety has been very common in Australia, and the appearance of two "humps" of temperature on the chart has caused it to be known as the "dromedary" type.

The signs and symptoms of the second or meningitic stage are as follows :—

1. The child is drowsy, but is irritable when disturbed.

2. Muscle twitchings and tremor may be noted.

3. There is complaint of pain in the neck and the "spinal sign" may be elicited. To obtain this sit the child up and try to persuade it to kiss its knee. It will try, but will give it up "because it hurts," *i.e.* because flexion of the spine gives rise to pain.

4. The sign of Amoss. This depends upon the fact that when the child sits up it likes to keep the spine extended, and to do this tends to lean backwards, supporting its weight on the extended arms, as on a tripod. If asked to fold the arms across the chest the child will probably do so, but quickly resumes its former position.

5. Kernig's sign may be present.

6. There is early loss of reflexes, especially in limbs which will later become paralysed.

7. Hyperæsthesia is commonly pronounced, either generally or in one or more limbs.

8. In addition, there are the signs of a generalized infection, such as sweating, fever, headache, and retention of urine.

If the above signs and symptoms are insufficient to enable a definite diagnosis to be made, they should at least suggest the imperative necessity for the performance of a diagnostic lumbar puncture. The cerebro-spinal fluid presents the following characters :—

It is under slightly increased tension. It is clear or slightly opalescent. The chlorides are normal in amount, whereas in tuberculous meningitis they are diminished. There is a variable increase in the cell count. At first this is mainly due to an increase in the polymorphonuclears, but later mononuclears predominate. As the paralytic stage approaches the globulin rapidly increases in amount.

It is quite possible to effect a diagnosis at this stage, though this is rarely done except in the presence of an epidemic. During the New York epidemic of 1931 57 per cent. of the cases are said to have been diagnosed in the preparalytic stage, and in Australia of late even a higher proportion have been so diagnosed. Seeing that this is the only time when specific treatment is of any value, it is to be hoped that we may be not less successful in this country, at least in the presence of an epidemic.

Paralysis does not, as a rule, appear for a week or more. The situation and extent are, of course, very variable. Any muscle may be affected, and may be completely paralysed or merely weakened. The latter condition is two and a half times as frequent as the former. This is what one would expect, since the vessels which supply the anterior horn cells run horizontally, and if any one of them were thrombosed it would give rise to a very limited destruction of the grey matter. Muscles, on the other hand, often derive their motor supply from quite an extensive area of the cord. Take, for example, the ilio-psoas. The nerve supply of this muscle is from the last dorsal and all the lumbar nerves, and it is rarely completely paralysed.

In every epidemic it has been noticed that when a case of anterior poliomyelitis crops up in a household other members of the family tend to present symptoms exactly similar to those of the preparalytic stage of poliomyelitis, but without themselves developing the illness. It has long been suspected that these were in reality abortive cases. Lovett examined a great many of them, taking careful measurements of their muscular power, and he found that a certain number did develop weakness of one or more muscles so slight that it would ordinarily escape observation. In many others muscular power was unaffected. In a case of the latter kind in the year 1910 Flexner injected the nasal washings into a monkey, with the result that this animal developed a typical attack of poliomyelitis. This observation has recently been repeated in two other cases by Paul and Trask.[1] Abortive cases in contacts are about ten times as frequent as are second cases of frank paralysis, and they probably form one of our chief sources of infection.

In addition to the common spinal type of paralysis other varieties are sometimes met with. In polioencephalitis we find an upper motor neuron lesion which produces a spastic paralysis, and when this is combined with spinal lesions we may encounter a mixture of spastic and flaccid paralyses. The cerebellum may be affected, producing nystagmus and ataxia; or the bulb, in which case death from respiratory failure is not uncommon. Landry's ascending paralysis has also been proved to be due to an infection with the virus of poliomyelitis.

Before proceeding to consider the further progress of the disease let us discuss the treatment of the acute stage, and first of all the use of serum obtained from convalescents. It has been proved experimentally that when immune serum and active virus are mixed in vitro and then injected into monkeys no disease is produced. If virus is injected intracerebrally and not later than twenty-four hours subsequently serum is administered either intraspinally or intravenously in sufficient quantity no disease follows, or at most a very modified attack after a prolonged incubation period.[2] If sufficient "convalescent serum" is administered, and the animal is inoculated with active virus a few days subsequently, again no disease will be produced. If, on the other hand, serum is administered after the onset of paralysis the disease follows its usual course and no benefit is demonstrable. It has also been discovered that a large proportion of the adult population becomes immunized to infection, and that the blood of such people contains bodies which are capable of conferring passive immunity on others.

During the last American epidemic attempts were made to immunize contact cases passively by injecting sera derived from adults. I believe that the results have been encouraging, though I have not been able to obtain any statistics in proof of this. All are agreed that when once paralysis has ensued serum treatment is useless, but opinions are by no means unanimous that it exercises any influence upon the course of the disease even when given in the preparalytic stage. Last year the New York Academy of Medicine expressed the view that the evidence both pro and con was still insufficient.[3] Dr. Jean Macnamara,[4] on the other hand, after studying the figures of cases treated with and without serum in Australia between the years 1925 and 1931 brings forward what appears to be conclusive evidence as to its value. It may be obtained free from the Lister Institute, and should undoubtedly be administered. In its absence it would be well worth while to give a mixed serum obtained from adults, preferably relatives.

It is usual to withdraw 20 to 30 cc. of cerebrospinal fluid, to have this rapidly examined, and if the result is positive to inject a similar amount of serum intrathecally through the same needle. Another 30 to 40 cc. should be given intravenously at the same time, and repeated if necessary next day.

If hypertonic saline is administered intravenously, it diminishes the tension of the cerebrospinal fluid, and lessens the œdema of the cord.

When given in conjunction with the serum it should, by reversing the direction of flow of the spinal fluid, bring the serum into more intimate contact with the virus which is present in the cord. Even by itself it most likely acts beneficially. Urotropine has been given in large doses as a disinfectant of the cerebrospinal fluid, but it is of doubtful value.

During the acute stage there is little else to do beyond keeping the patient quiet and warm and making sure that all the paralysed muscles are maintained in a state of relaxation. This last is most important, for serious deformities may arise during the first few weeks. One should be particularly on guard to prevent footdrop; flexion of knee and hip, if the child is allowed to lie on one side or to sit up; and spinal curvature caused by weakness of the spinal or abdominal muscles. The error is not infrequently made of examining the limbs only, and forgetting all about the muscles of the trunk till gross deformity has taken place. The most generally useful form of splint is the Thomas's double abduction frame with footpieces, and if necessary with armpieces added or, if there is abdominal weakness, a canvas corset. Each case is an individual problem, which is what makes poliomyelitis such an interesting disease to treat.

It is only necessary to visualize the inflamed and hæmorrhagic spinal cord to realize how much harm can be done at this stage by misguided massage, electrical stimulation, or, in fact, any form of active treatment. Rest and an absence of stimuli is what the cord needs for its recovery, and this can best be ensured by complete rest of the body as a whole. The question naturally arises: "When can one start upon active measures?" The answer is: "Not while hyperæsthesia is present," or in the absence of that symptom: "Not for the first six to eight weeks." During this period regard the child as potentially infectious. Keep other children away as much as possible. Regard adults as possible carriers. It is probable that they should spray their noses with some weak antiseptic, such as pot. permang., which is known to have a pronounced viricidal action. During an epidemic it may be necessary to close schools and to keep children away from cinemas, churches and other places of instruction or entertainment, but if an outbreak occurs in a boarding-school it is a mistake to close the school and send the children home to infect their brothers and sisters all over the country.

When the acute stage is over comes the period of convalescence, and this may be regarded as lasting for at least the next two or three years. This is perhaps the most important period, for during it so much good and so much harm may be done.

Let us consider the harm first.

Muscles which have been merely weakened may be completely paralysed either by fatigue or by stretching. Their unparalysed opponents exercise a constant pull if they are allowed to do so. In so doing they themselves will become shortened and less efficient. Paralysed muscles, then, must be kept continuously relaxed throughout this period if good results are to be obtained.

Now as to fatigue. It is generally good to let children get about, but in so doing they are very apt to throw undue strain upon weakened muscles, and they therefore require most careful supervision. A chart recording all the paralysed and weakened muscles should be kept and brought up to date at regular intervals. In this way only can one be certain whether progress is satisfactory and adapt one's treatment accordingly. Positive treatment consists mainly in splinting and in the careful training of weakened and paralysed muscles. Heat and massage are useful for promoting the circulation, but it is on active voluntary movement that one must chiefly rely. This promotes the development of fresh paths by which impulses may pass down from the brain, and is the natural way in which one would expect muscular development to take place.

There are, however, numerous precautions to be taken.

Firstly, things must be made very easy for the feeble muscles.

The influence of gravity is eliminated by various devices, *e.g.* the patient may exercise in a warm bath; all movements may be carried out in a horizontal plane, as upon a slippery table or with the limb supported in a sling suspended from a gallows. The movement of a limb should be assisted at the beginning and end of its range, at which points most effort is required. At the slightest sign of fatigue the exercise must be stopped. In many cases it will be found that two or three sittings a week are more useful than are daily ones.

Our great objects throughout this period are to encourage function and to prevent deformity. Examinations of the patient should be frequent and thorough. The child should be stripped, so that one may at once detect any deformity and correct it while this can still be easily done. The gait, too, must be noticed and any abnormality carefully analysed to make certain of its origin.

After two or three years comes the time when one must realize that little more improvement can be expected except as the result of surgical measures. Every case is a problem to itself, as it is rare to find any two which are alike in the distribution and extent of their paralyses. Usually one has to make up one's mind whether the needs of the patient will best be met by the employment of apparatus alone, whether operation will enable us to dispense with apparatus altogether, or whether a combination of the two will give the best functional result.

It would be foolish for me to attempt any account of the operative surgery of anterior poliomyelitis. Speaking broadly, we aim first at the correction of deformities by manipulations and plasters or by operations upon the soft parts or bones; secondly, at the stabilization of flail joints; and thirdly, at the transplantation of tendons in order to make a healthy muscle take the place of a weakened one. Fixation of joints, notably those of the foot, the knee, and the shoulder, sometimes yields the most brilliant results. Tendon transplantation has a more limited field, but in selected cases the results are very satisfactory, especially when combined with a fixation operation.

I have so far said nothing of the trophic changes which are a prominent feature of the disease. The growth of limbs is stunted. Hands and feet are cold and, in winter especially, covered in chilblains. Painful corns and ulcers are by no means uncommon. Long woollen garments and the avoidance of any constriction of the limbs, especially by tight boots or by the straps of apparatus, help matters. Sinusoidal baths given about twice weekly are excellent for keeping chilblains in check, but I believe that there is a great future in these cases for operations upon the sympathetic system. Leriche's operation, which consists in stripping off the sheaths of the large vessels of a limb, together with the sympathetic fibres which they contain, causes a temporary dilatation of the vessels distal to the site of operation, but the effect is only temporary. Removal of the lumbar sympathetic ganglia, on the other hand, causes a dilatation of the vessels of the extremity, which lasts, as I know from personal experience, for several years, and which I believe to be permanent. In the case of the upper limb the same effect is produced by removal of the upper dorsal and lower sympathetic ganglia. It is quite likely that our chief surgical advances in the treatment of these cases will lie along these lines.

Lastly, I want to say a few words about after-care. Much of our work is wasted by lack of an efficient follow-up system. This is especially the case where families migrate, and I do feel that something should be done to see that every crippled child is transferred with its complete dossier to the nearest orthopædic centre whenever it moves from one district to another. A good deal is already being done to provide vocational training. It needs to be carried out on a large scale to be truly effective. I am glad to know that a scheme is on foot to found a centre for the whole of the West of England at Exeter. Where there are enough cases it is possible to supply a big range of different types of training, and also to find openings for the pupils when trained. At Exeter there is to be an employment bureau.

Employment is always the greatest difficulty. Even if a cripple does a job as well as a normal individual he seldom gets it in open competition. I believe that employers make a great mistake in this respect, for it has been shown over and over again that cripples stick to their jobs better than the healthy. Their work means so much more to them. It is the chief interest of their lives, since they are debarred from so much outside. I dare say, too, that they feel that a job once lost may never be replaced by another. In this mechanical age factory work can often be done perfectly well by one with a single arm, or who has two arms but no legs. At any rate, there must often be a few such jobs in a factory. Henry Ford, a pioneer in this as in so many other ways, gives work of this type to the maimed, but he is the only employer, to the best of my knowledge, who does so.

In conclusion, although I cannot claim much originality for the subject-matter of this paper, I trust that it may serve to stimulate interest in a disease which may at any time become of very vital importance to the whole nation.

### REFERENCES.

[1] Paul, J. R., and Trask, J. D., *Jour. Exper. Med.*, 1932, lvi. 319.

[2] Flexner, S., and Lewis, P. A., *Jour. Amer. Med. Assoc.*, 1910, liv. 1,780.

[3] Park, W. H., *Jour. Amer. Med. Assoc.*, 1932, xcix. 1,050.

[4] Macnamara, J., and Morgan, F. G., *Lancet*, 1932, i. 469.

